# Non-DNA binding, dominant-negative, human PPARγ mutations cause lipodystrophic insulin resistance

**DOI:** 10.1016/j.cmet.2006.09.003

**Published:** 2006-10

**Authors:** Maura Agostini, Erik Schoenmakers, Catherine Mitchell, Istvan Szatmari, David Savage, Aaron Smith, Odelia Rajanayagam, Robert Semple, Jian'an Luan, Louise Bath, Anthony Zalin, Mourad Labib, Sudhesh Kumar, Helen Simpson, Dirk Blom, David Marais, John Schwabe, Inês Barroso, Richard Trembath, Nicholas Wareham, Laszlo Nagy, Mark Gurnell, Stephen O'Rahilly, Krishna Chatterjee

**Affiliations:** 1 Department of Medicine, University of Cambridge, United Kingdom; 2 Department of Clinical Biochemistry, University of Cambridge, United Kingdom; 3 Department of Biochemistry and Molecular Biology, University of Debrecen, Hungary; 4 Medical Research Council Epidemiology Unit, Cambridge, United Kingdom; 5 Royal Hospital for Sick Children, Edinburgh, United Kingdom; 6 Wordsley Hospital, Stourbridge, United Kingdom; 7 Department of Medicine, University of Warwick, Coventry, United Kingdom; 8 Department of Internal Medicine, University of Cape Town, South Africa; 9 Medical Research Council, Laboratory of Molecular Biology, Cambridge, United Kingdom; 10 Metabolic Disease Group, Wellcome Trust Sanger Institute, Cambridgeshire, United Kingdom; 11 Department of Medical and Molecular Genetics, King's College, London, United Kingdom

## Abstract

PPARγ is essential for adipogenesis and metabolic homeostasis. We describe mutations in the DNA and ligand binding domains of human PPARγ in lipodystrophic, severe insulin resistance. These receptor mutants lack DNA binding and transcriptional activity but can translocate to the nucleus, interact with PPARγ coactivators and inhibit coexpressed wild-type receptor. Expression of PPARγ target genes is markedly attenuated in mutation-containing versus receptor haploinsufficent primary cells, indicating that such dominant-negative inhibition operates in vivo. Our observations suggest that these mutants restrict wild-type PPARγ action via a non-DNA binding, transcriptional interference mechanism, which may involve sequestration of functionally limiting coactivators.

## Introduction

The nuclear receptor (NR) peroxisome proliferator-activated receptor γ (PPARγ) is a ligand-inducible transcription factor that is essential for adipocyte differentiation (Tontonoz et al., 1994b; Barak et al., 1999; Rosen et al., 1999). Alternative splicing and differential promoter usage generates two protein isoforms: PPARγ2, expressed from a single γ2 promoter, contains an additional 28 amino-terminal amino acids and is nearly adipose-specific; PPARγ1, whose expression can be regulated by multiple (γ1, γ3, γ4) promoters, is more ubiquitously distributed. In addition to adipogenesis, PPARγ also plays an important role in adipocyte lipid metabolism, regulating target genes (lipoprotein lipase, fatty-acid transport protein, aquaporin) that mediate triglyceride hydrolysis and fatty acid and glycerol uptake, together with genes (acylCoA synthetase, PEPCK, glycerol kinase) involved in fatty acid re-esterification and lipid storage (Lehrke and Lazar, 2005; Savage, 2005). The thiazolidinedione (TZD) class of antidiabetic agents are synthetic, high-affinity PPARγ ligands (Lehmann et al., 1995) and putative endogenous activators include fatty acids, eicosanoids, and prostaglandin derivatives (Desvergne and Wahli, 1999) as well as undefined ligands produced during adipocyte differentiation (Tzameli et al., 2004).

The most common population genetic variant of PPARγ is a polymorphism replacing alanine for proline at codon 12 (Pro12Ala) in PPARγ2, with a meta-analysis of association studies showing that the Pro allele confers a modest but significant increase in diabetes risk (Altshuler et al., 2000). The discovery that PPARγ is a target for TZDs, which act by enhancing tissue insulin sensitivity, prompted screening of a cohort of subjects with severe insulin resistance, with identification of two missense PPARγ mutations (P467L, V290M) in unrelated cases (Barroso et al., 1999). Functional studies showed that these mutant receptors retain DNA binding but exhibit significant impairment of transcriptional activation and coactivator recruitment in response to different ligands (Barroso et al., 1999; Agostini et al., 2004), due to the mutations destabilizing the carboxyterminal α helix of PPARγ (Kallenberger et al., 2003), which mediates these functions. Consonant with heterozygosity in affected subjects and dominant inheritance in one kindred, the P467L and V290M mutant receptors inhibited the transcriptional activity of wild-type (WT) PPARγ in a dominant-negative manner (Barroso et al., 1999). Subsequently, two further heterozygous mutations in the ligand binding domain (LBD) of PPARγ (R425C; F388L) have been described, with recognition that in addition to insulin resistance the phenotype also includes a stereotyped pattern of partial lipodystrophy (PLD) (Hegele et al., 2002; Agarwal and Garg, 2002; Savage et al., 2003).

Following this, we described several individuals who were heterozygous for a frameshift/premature stop codon mutation, ([A^553^ΔAAAiT]fs.185[stop186]-hereafter abbreviated to FSX) in the DNA binding domain (DBD) of PPARγ, with this truncation mutant lacking DNA binding, transcriptional, and dominant-negative activity. Significantly, heterozygosity for the FSX mutation alone was not associated with insulin resistance, but individuals who were doubly heterozygous, with an additional defect in an unrelated gene encoding the muscle-specific regulatory subunit of protein phosphatase 1 (PPP1R3A), exhibited severe insulin resistance (Savage et al., 2002). Heterozygosity for a single nucleotide substitution in the promoter of human PPARγ4 leading to its altered expression in vitro has been associated with PLD and insulin resistance in one family, but the authors did not exclude the possibility of interaction with a defect at a second genetic locus to produce this phenotype (Al-Shali et al., 2004).

Here, we describe the identification of five heterozygous human PPARγ mutations (C114R, C131Y, C162W, R357X, [A^935^ΔC]fs.312[stop315]-hereafter abbreviated to FS315X) not associated with a PPP1R3A gene defect, in unrelated cases of lipodystrophic insulin resistance and show that these mutants inhibit WT receptor action via a non-DNA binding, dominant-negative mechanism.

## Results and Discussion

### Heterozygous PPARγ mutations are associated with lipodystrophic insulin resistance

The case histories (see the Supplemental Data available with this article online) and characterization (Table 1) of index subjects (S1–S5) harboring PPARγ mutations indicate many of the features associated with previously described cases (Barroso et al., 1999; Hegele et al., 2002; Agarwal and Garg, 2002; Savage et al., 2003). All subjects showed marked fasting hyperinsulinaemia (Table 1) with acanthosis nigricans in a subset (S3, S4, S5), denoting severe insulin resistance; total body fat was reduced in all individuals, and imaging indicated a stereotyped pattern of partial lipodystrophy affecting gluteal (Figure S1) and peripheral limb depots; hepatic steatosis and marked dyslipidaemia (raised triglycerides, low high-density lipoprotein cholesterol [HDL-C]) with secondary complications (cutaneous eruptive xanthomata S3, S4; pancreatitis S5) were features of all cases; several individuals (S2, S3, S5) exhibited early-onset hypertension.

We sequenced the γ4 promoter, coding exons and splice junctions of *PPARG* and identified heterozygous, missense mutations in the DBD (S1–S3), or premature stop mutations in the LBD (S4, S5) of the receptor in index cases. *PPARG* has also been sequenced by us in 215 additional subjects, comprising 93 patients from our severe insulin resistance cohort (Barroso et al., 1999), 48 CEPH individuals of European descent and 27 Europid, hyperinsulinaemic participants in the Ely study (Williams et al., 1995), and 47 controls from four different ethnic groups, or sequenced by others in 24 African and 23 CEPH European individuals (Seattle SNPs project, http://pga.gs.washington.edu), and other than the Pro12Ala polymorphism neither these or other mutations have been identified. We also sequenced *PPP1R3A* in each proband and identified no mutations or polymorphisms, excluding a second genetic defect at this locus as described previously (Savage et al., 2002).

**Figure 1:**
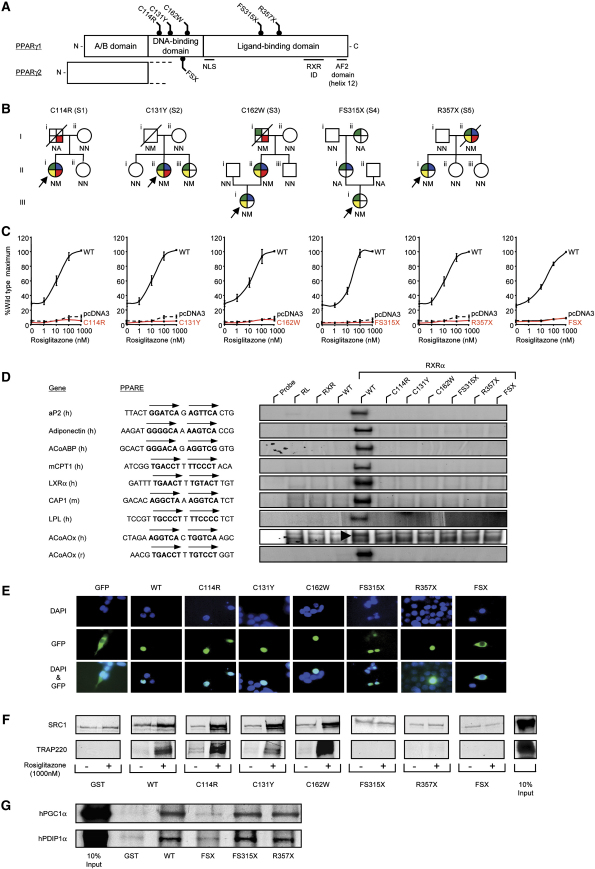
Identification and characterization of loss-of-function mutations in human PPARγ **A)** Schematic representation of the three major domains of PPARγ, showing the locations of the five mutations (C114R, C131Y, C162W, FS315X, and R357X – PPARγ1 nomenclature) and the previously reported FSX mutation. NLS, nuclear localisation signal; RXR ID, retinoid X receptor interaction domain; AF2, activation function 2 domain. **B)** Family pedigrees showing genotypes (N, wild-type allele; M, mutant allele; NA, not available) and phenotypes (colored segments denote the presence of specific traits: green, type 2 diabetes mellitus/impaired glucose tolerance/hyperinsulinaemia; yellow, hypertriglyceridaemia; blue, hypertension; red, ischemic heart disease). Squares and circles represent male and female family members; slashed symbols denote deceased family members and arrows denote probands. **C)** PPARγ mutants are unable to mediate ligand-dependent transactivation. 293EBNA cells were transfected with 100 ng of wild-type (WT), mutant, or empty (pcDNA3) expression vectors, together with 500 ng of (PPARE)_3_TKLUC reporter construct and 100 ng of Bos-β-gal internal control plasmid, and increasing concentrations of rosiglitazone. Results are expressed as a percentage of the maximum activation with WT PPARγ1 and represent the mean ± SEM of at least three independent experiments in triplicate. **D)** PPARγ mutants are unable to bind to DNA. EMSA with in vitro translated wild-type (WT) or mutant PPARγ1 (C114R, C131Y, C162W, FS315X, R357X, or FSX) and RXR proteins coincubated with oligonucleotide duplexes corresponding to various natural PPAREs. aP2, adipocyte protein 2; ACoABP, acyl coenzyme A binding protein; mCPT1, muscle carnitine palmitoyl transferase 1; LXRα, liver X receptor α; CAP, cbl-associated protein; LPL, lipoprotein lipase, ACoAOx, acyl coenzyme A oxidase; h, human; m, mouse; r, rat; RL, reticulocyte lysate. **E)** The C114R, C131Y, C162W, FS315X, and R357X mutants translocate to the nucleus whereas the FSX mutant remains cytoplasmic. 293EBNA cells were transfected as described. Top panels show DAPI-staining (blue) of nuclei, middle panels the cellular localisation of GFP-tagged receptors, and lower panels merged images. **F)** The DBD PPARγ mutants recruit SRC1 and TRAP220 coactivators, whereas the FS315X, R357X, and FSX truncation mutants do not interact. GST alone or WT and mutant GST-PPARγ fusion proteins were tested with ^35^S-labeled in vitro translated SRC1 (upper panel) or TRAP220 (lower panel) in the absence or presence of rosiglitazone. Coomassie-stained gels confirmed comparable protein loading (data not shown). **G)** The LBD truncation mutants (FS315X, R357X) recruit PGC1α and PDIP1α coactivators, whereas the FSX mutant fails to interact. GST alone or WT and mutant GST-PPARγ fusion proteins were tested with ^35^S-labeled in vitro translated human PGC1α and human PDIP1α in the absence of ligand. Coomassie-stained gels confirmed comparable protein loading (data not shown).

Heterozygosity for PPARγ mutations in a parent and grandparent of S3 and a parent of S5 segregated with phenotype, constituting a dominant inheritance pattern in two families; one sibling of S2 with dyslipidaemia and insulin resistance was heterozygous for the PPARγ mutation whereas another genetically unaffected sibling was biochemically normal; the ascertainable family members of S1 were unaffected and normal and no relatives of S4 could be contacted (Figure 1B).

### PPARγ mutants fail to bind DNA and are transcriptionally inactive

Three missense mutations involve highly conserved cysteine residues within (C114R, C131Y, C162W) the DBD and two further nonsense (R357X) or frameshift/premature stop (FS315X) mutations truncate the receptor within the central part of its LBD (Figure 1A), predicting loss-of-function of the mutant proteins. We therefore characterised and compared the properties of these PPARγ mutants with the FSX mutant described previously (Savage et al., 2002).

The receptor mutants exhibited negligible transcriptional activity, lacking constitutive basal activity noted previously with WT PPARγ (Agostini et al., 2004; Zamir et al., 1997) as well as any response to rosiglitazone, a TZD receptor agonist (Figure 1C). Such complete loss of function was similar to the FSX mutant and might be anticipated with analogous truncation mutants (FS315X, R357X) not possessing the transactivation (AF2) domain at the receptor carboxyterminus (Figure 1A) (Zamir et al., 1997; Wu et al., 2003), but the lack of function with DBD mutants (C114R, C131Y, C162W), prompted further investigation of their DNA binding properties.

PPARγ heterodimerizes with the retinoid X receptor (RXR) and this complex has been shown to bind a DNA response element (PPARE), consisting of a direct repeat (DR1) of the consensus sequence (AGGTCA) separated by a single nucleotide (Ijpenberg et al., 1997) and a recent study has suggested that the stringency of PPARγ binding to some response elements is relatively relaxed, not needing complete integrity of its DBD (Temple et al., 2005). A range of previously documented or predicted PPAREs from known target genes were therefore tested in electrophoretic mobility shift assays and both DBD and LBD truncation receptor mutants showed negligible heterodimeric binding (Figure 1D). To examine interaction of mutant receptors with RXR, we coexpressed VP16-full length PPARγ fusions with Gal4DBD-RXR in a mammalian two-hybrid assay. In keeping with preservation of the dimerization interface (Gampe et al., 2000) within their intact LBD (Figure 1A), the DBD mutants interacted readily whereas the FS315X, R357X, and FSX mutants lacking this interface failed to be recruited to Gal4-RXR (Figure S2). It was therefore conceivable that the DBD mutants could be recruited indirectly to a PPARE by binding RXR (Gampe et al., 2000), or conversely, that the LBD truncation mutants might bind a PPARE monomerically as has been documented with the thyroid hormone receptor (TR) (Lazar et al., 1991). However, unlike WT receptor, VP16-full length, mutant PPARγ fusions were unable to activate a PPARE-containing reporter gene (Figure S3), indicating that like FSX, these PPARγ mutants do not bind DNA directly or indirectly.

### PPARγ mutants translocate to the nucleus and interact with cofactors

The intracellular localization of WT PPARγ is predominantly nuclear (Akiyama et al., 2002) and, analogous to steroid/thyroid hormone receptors, may be dependent on a putative nuclear localisation signal (NLS) located between its DBD and LBD (Figure 1A) (Guiochon-Mantel and Milgrom, 1993; Zhu et al., 1998). Studies of GFP- PPARγ fusions showed that, in keeping with preservation of the putative NLS, both DBD and LBD truncation mutants localized to the nucleus comparably to WT, whereas the FSX truncation mutant, which lacks this sequence, remained cytoplasmic similar to GFP alone (Figure 1E).

We next examined whether the PPARγ mutants might also retain the ability to interact with transcriptional coactivators. Steroid receptor coactivator-1 (SRC1/NCoA1) (Onate et al., 1995) and PPARγ binding protein/thyroid receptor-associated protein 220 (PBP/TRAP220) interact directly with the AF2 domain of PPARγ, with the latter cofactor being required for receptor-mediated adipogenesis (Zhu et al., 1996, 1997; Ge et al., 2002). Consistent with preservation of their AF2 domains, protein-protein interaction assays showed ligand-dependent binding of SRC1 or TRAP220 to the DBD mutants, but no specific interaction with FSX or LBD truncation mutants, which lack this region (Figure 1F). Conversely, we hypothesized that the PPARγ LBD truncation mutants would retain the ability to recruit coactivators, which can interact with receptor independently of its AF2 domain. PPARγ coactivator-1 (PGC1), which augments receptor action in fat cells (Puigserver and Spiegelman, 2003), can bind PPARγ via its DBD and hinge region (αα 128-229) (Puigserver et al., 1999); PDIP, isolated in a two hybrid assay using the DBD/hinge region of PPARγ (Tomaru et al., 2006), is a coactivator that also enhances PPARα activity (Surapureddi et al., 2002). Both PGC1 and PDIP bound WT or FS315X and R357X LBD truncation mutants in protein-protein interaction assays, whereas the FSX mutant showed negligible interaction (Figure 1G).

### PPARγ signaling is reduced in mutation-containing primary cells ex vivo or mutant-expressing cells in vitro

**Figure 2:**
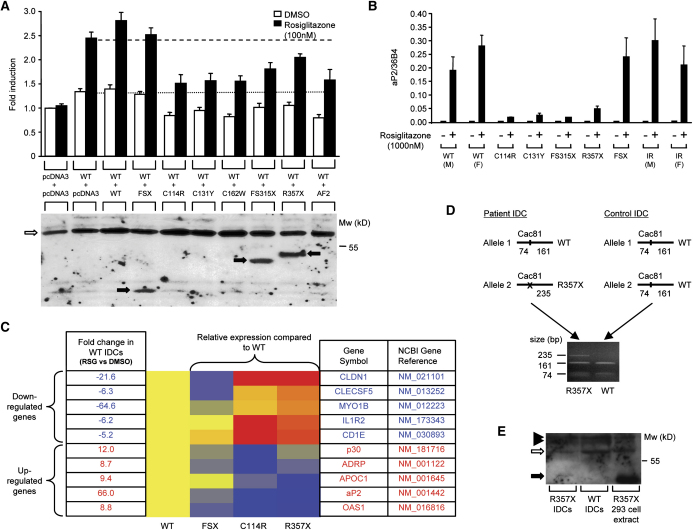
PPARγ mutants exhibit dominant-negative activity **A)** The C114R, C131Y, C162W, FS315X, and R357X PPARγ mutants inhibit transactivation by wild-type (WT) receptor, comparably to AF2, an artificial PPARγ mutant described previously, whereas the FSX mutant lacks dominant-negative activity (upper panel). 3T3-L1 cells were cotransfected with 33 ng of WT receptor plus an equal amount of either empty (pcDNA3) or WT or mutant expression vector, together with 265 ng of human aP2LUC reporter plasmid and 65 ng of the internal control plasmid Bos-β-gal. The dotted and dashed lines denote transcriptional activity of WT receptor in the absence and presence of ligand respectively. Results are expessed as fold induction relative to empty vector (pcDNA3 + pcDNA3) and represent the mean ± SEM of at least three independent experiments in triplicate. Expression of wild-type and mutant receptor proteins was confirmed by Western blotting (lower panel) and the positions of WT, C114R, C131Y, C162W, and AF2 PPARγ (open arrow) and FSX, FS315X and R357X truncation mutants (solid arrows) are indicated. **B and C)** Ligand-dependent regulation of PPARγ target genes in IDCs from subjects with PPARγ mutations. (**B**) Induction of the aP2 gene by rosiglitazone, measured by qPCR, is markedly impaired in IDCs derived from subjects with the C114R, C131Y, FS315X, and R357X mutations, compared to responses in cells from normal (WT), severely insulin resistant (IR) subjects without mutations in PPARγ and cells with the FSX, haploinsufficient, mutation. Results represent the mean ± SEM of more than three independent experiments in triplicate, except for cells with the FS315X mutation where a single representative experiment is shown. (**C**) Relative expression of several PPARγ target genes (5 downregulated and 5 upregulated) in WT and mutation-containing (FSX, C114R, R357X) IDCs, measured by qPCR using TLDA. Red indicates higher, and blue lower, levels of gene expression relative to rosiglitazone-treated (1000 nM) WT cells, whose responses are uniformly designated yellow. Fold changes in expression of each gene in rosiglitazone (RSG) versus vehicle (DMSO) treated WT cells are also listed. **D and E)** The R357X PPARγ mutant is expressed in IDCs. (**D**) PPARγ cDNA flanking the R357 codon was amplified by RT-PCR in IDCs from patient S5 and a control subject. Cac81 enzyme digestion of PCR products derived from the WT allele yields two fragments (161 and 74 bp), whereas abolition of this restriction site in the R357X mutant allele yields a larger 235 bp product. (**E**) Whole-cell lysates of WT and R357X mutant IDCs and 293EBNA cells transfected with R357X mutant were immunoprecipitated and Western blotted. The positions of WT PPARγ (open arrow), R357X (solid arrow), and nonspecific bands (solid arrowheads) are indicated.

The observation that these PPARγ mutants translocate to the nucleus and interact with coactivators raised the possibility that they might interfere with WT receptor signaling. The murine adipocyte P2 (aP2) gene is a classical target of PPARγ action (Tontonoz et al., 1994a; Guan et al., 2005) and the human homolog (FABP4) is similarly responsive (Pelton et al., 1999). When coexpressed with WT PPARγ at equivalent levels in 3T3-L1 adipocytes, the DBD and LBD mutants blocked WT receptor mediated activation of the human aP2/FABP4 gene promoter comparably to an artificial, dominant-negative PPARγ mutant (AF2) described previously (Gurnell et al., 2000), whereas FSX lacked dominant-negative inhibitory activity (Figure 2A).

We wished to determine whether such divergent dominant-negative inhibition by these PPARγ mutants versus FSX might operate in vivo. PPARγ is highly expressed in immature dendritic cells (IDCs) derived from primary human blood monocytes and mediates marked receptor responsiveness, with strong ligand-dependent induction of aP2 expression in these cells (Szatmari et al., 2004). Induction of aP2/FABP4 expression in IDCs containing DBD or LBD PPARγ mutations was severely attenuated compared to responses in control cells from either normal individuals (WT) or from subjects (IR) with comparable insulin resistance without a PPARγ gene defect. Significantly, aP2 induction in FSX mutation-containing cells was comparable to responses from control subjects (Figure 2B). We examined other PPARγ target genes, identified from extensive microarray profiling of normal IDCs (I.S. and L.N., unpublished data) and found that responses to PPARγ agonist in DBD and LBD truncation mutation-containing cells were markedly attenuated whereas FSX mutation-containing cells exhibited responses that were either similar or only slightly reduced compared to WT cells (Figure 2C). PPARγ mRNA levels in control and mutation-containing primary cells were similar (data not shown), suggesting that differential responsiveness was not due to altered receptor expression. Furthermore, PPARγ mRNA from both WT and R357X alleles was expressed in mutation-containing IDCs (Figure 2D), indicating that the R357X transcript is not subject to nonsense-mediated decay (Culbertson, 1999) and both WT and R357X mutant PPARγ proteins were also expressed in these cells (Figure 2E).

**Figure 3:**
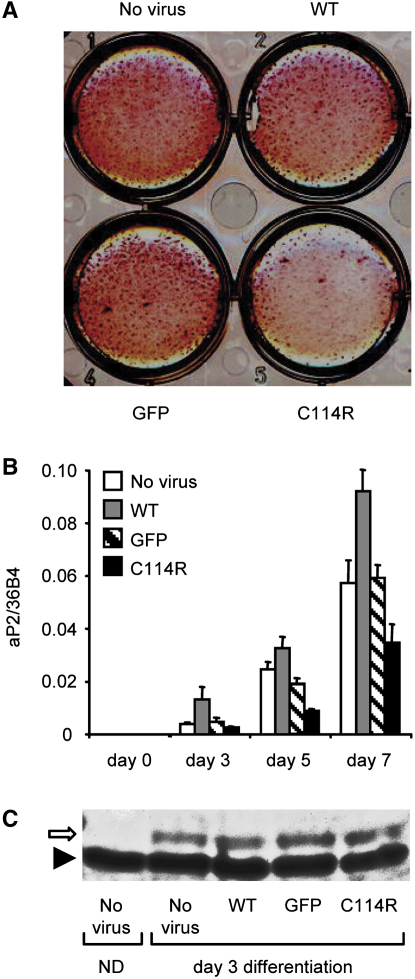
Adenoviral-mediated expression of the C114R PPARγ mutant inhibits human preadipocyte differentiation Chub-S7 human preadipocyte cells were infected with comparable efficiency using recombinant adenoviruses expressing GFP, GFP-WT, or GFP-C114R mutant PPARγ and differentiated in the presence of rosiglitazone (100nM). **A)** Fully differentiated Chub-S7 cells fixed and stained with oil red O. **B)** aP2 expression quantitated by real-time qPCR at days 0, 3, 5, and 7 postdifferentiation with results representing the mean ± SEM of at least three independent experiments in triplicate. **C)** Western blotting of Chub-S7 cells at day 4 posttransduction with recombinant adenoviruses confirming comparable levels of total receptor expression. Nondifferentiated, (day 0) nontransduced, cell extracts (ND) are shown for comparison. The positions of PPARγ (open arrow) and nonspecific band (solid arrowhead) are indicated.

Finally, we determined whether dominant-negative inhibition by a non-DNA binding PPARγ mutant could interfere with a receptor-mediated biological process. Compared to control, WT PPARγ or GFP adenovirus-transduced human preadipocyte cells, both cellular differentiation (Figure 3A) and aP2 gene expression (Figure 3B) in cells transduced with C114R mutant PPARγ adenovirus were significantly reduced.

### Transcriptional interference via a non-DNA binding mechanism

We have shown previously that dominant-negative inhibition by PPARγ mutants (P467L, V290M), is mediated by repression of target genes by DNA-bound mutant receptors, analogous to mechanisms of other mutant nuclear receptors (e.g., the v-erbA oncogene, TRβ mutants in Resistance to Thyroid Hormone, PZLF-RARα fusion proteins in promyelocytic leukaemia) (Love et al., 2000). In contrast, the missense DBD and LBD truncation mutants identified here are unable to bind DNA, yet can inhibit WT PPARγ action, suggesting a different mechanism of transcriptional interference. Competition for shared cofactors by NRs was postulated to explain mutual antagonism of progesterone and estrogen receptor signaling (Meyer et al., 1989) and the subsequent observation that SRC1, a shared coactivator, could relieve such “squelching”, validated this hypothesis (Onate et al., 1995). Ligand-activated NRs have been shown to inhibit either their own function (Barettino et al., 1994) or that of heterologous receptors (Zhang et al., 1996) by limiting the availability of coactivators that are recruited to their transactivation domains. Our observations indicate that non-DNA binding, dominant-negative PPARγ mutants can recruit coactivators, suggesting an analogous cofactor sequestration mechanism for thereby restricting WT receptor function. Evidence suggests that similar mechanisms operate to inhibit PPAR signaling in other contexts: analogous to our natural DBD mutants, others have generated artificial, dominant-negative, PPARγ DBD mutants, which block either adipogenesis (Park et al., 2003) or neural stem cell differentiation (Wada et al., 2006); γORF4 is a newly identified human PPARγ splice variant with a truncated LBD (αα273), which has dominant-negative activity and is selectively overexpressed in colorectal neoplasia (Sabatino et al., 2005); a dominant-negative PPARα splice variant with a truncated LBD (αα 174), is expressed in human tissues including liver (Gervois et al., 1999). Interestingly, heterozygous, non-DNA binding mutations in some nuclear receptors do not mediate a phenotype: mutations in the DBD of VDR only cause vitamin D resistance in the homozygous state (Malloy et al., 1999); a “knock-in” mutation in the DBD of murine TRβ does not produce thyroid hormone resistance (Shibusawa et al., 2003). Possibly due to its pivotal role in regulating transcription of genes mediating both adipocyte formation and function (Lehrke and Lazar, 2005), we suggest that PPARγ signaling may be particularly sensitive to interference via the postulated “squelching” mechanism, with deleterious metabolic consequences. A corollary of this may be that even modest enhancement of normal receptor activity in key tissues could be beneficial, supporting attempts to develop partial or tissue-specific PPARγ agonists (Reginato et al., 1998; Rocchi et al., 2001; Berger et al., 2003).

## Experimental procedures

### Sequencing of PPARγ and PPP1R3A genes

The PPP1R3A (exons 1-4) and PPARγ (exons 1-6, B and promoter region of PPARγ4) genes were amplified using specific primers (available upon request) and sequenced as decribed previously (Savage et al., 2002).

### Construction of PPARγ mutants and other vectors

Full length WT and mutant PPARγ1 cDNAs were cloned in pGEX4T (Amersham Pharmacia Biotech), pCMX-VP16 (kind gift from R. Evans), pSG424 (Sadowski and Ptashne, 1989) and pEGFP-C1 (Clontech), to yield GST-PPARγ1, VP16-PPARγ1, Gal4DBD-PPARγ and GFP-PPARγ1 fusions respectively.

### Electrophoretic mobility shift assays

Electrophoretic mobility shift assays (EMSA) were performed as described (Collingwood et al., 1994) with different natural PPAREs: aP2, derived by alignment of human and murine promoter sequences (Graves et al., 1992); Adiponectin (Iwaki et al., 2003): ACoABP (Helledie et al., 2002); mCPT1, (Mascaro et al., 1998); LXRα, (Laffitte et al., 2001); CAP1, (Baumann et al., 2000); LPL, (Schoonjans et al., 1996); ACoAOx, (Varanasi et al., 1996); ACoAOx (Zamir et al., 1997).

### Transfection assays

293EBNA cells, cultured in DMEM/10%FCS were transfected with Lipofectamine2000- or calcium phosphate-mediated in 96- or 24-well plates respectively and assayed for luciferase and β-galactosidase activity as described (Collingwood et al., 1994) following 36 hr with or without ligand. 3T3-L1 adipocyte cells were cultured and transfected with Lipofectamine2000 in 24-well plates as described above.

### Cellular localisation of EGFP-tagged mutants

293EBNA cells, grown on glass well slides were transfected using Lipofectamine 2000 with 1μg of EGFP-PPARγ1 fusions, fixed with 4% paraformaldehyde, mounted using vectashield and fluorescence was visualized by digital microscopy.

### Peripheral blood monocyte purification and IDC culture

With ethical approval, monocytes were harvested from peripheral blood by Ficoll gradient centrifugation and immunomagnetic cell separation using anti-CD14-conjugated microbeads (VarioMACS; Miltenyi Biotec), resuspended in 6-well plates at a density of 1.5 × 10^6^ cells/ml and cultured in RPMI 1640 plus 10% FBS containing 800U/ml GM-CSF (Leucomax) and 500U/ml IL-4 (Peprotech) to generate IDCs as described (Sallusto and Lanzavecchia, 1994) with or without exposure to ligand for 24 hr.

### Quantitative real-time PCR analysis of gene expression

100ng of total RNA from IDCs, isolated using TRIZOL (Invitrogen), was reverse transcribed and analyzed by Taqman quantitative real-time PCR (qPCR) as described (Szatmari et al., 2004). The sequences of primers and probes are available upon request.

Taqman qPCR low density arrays (TLDA) were used to quantify the expression of multiple target genes in IDCs, according to the manufacturer's instructions.

To obtain cDNA, RNA was reverse transcribed using a High Capacity cDNA Archive kit (Applied Biosystems). The following commercially available Taqman assays (Applied Biosystems) were used: ADRP/ADFP (Hs00605340_m1), APOC1 (Hs00155790_m1), CLDN1 (Hs00221623_m1), aP2/FABP4 (Hs00609791_m1), CLECSF5 (Hs00183780_m1), CD1E (Hs00229421_m1), MYO1B (Hs00362654_m1), IL1R2 (Hs00174759_m1), OAS1 (Hs00242943_m1), p30 (Hs00396457_m1), cyclophilinA/PPIA (Hs99999904_m1). The comparative Ct method was used to quantify transcripts and normalize to cyclophilinA expression levels, which did not vary with ligand treatment. Thereafter, data were further normalized to expression levels in ligand-treated WT IDC samples using GeneSpring 7.2 software (Agilent).

### RFLP analysis of PPARγ transcripts

PPARγ cDNAs were amplified from WT or R357X mutation-containing IDCs by RT-PCR using forward (CTCCTTGATGAATAAAGATGGGG) and reverse (ATGTCTTCAATGGGCTTCACAT) primers, the PCR products were digested with Cac8I enzyme (New England Biolabs) and analyzed by agarose gel electrophoresis.

### Immunoprecipitation and Western blot analysis

IDCs, harvested from 200ml of peripheral blood, were lysed in RIPA buffer containing a protease inhibitor cocktail (Roche) and cell supernatants immunoprecipitated using a mouse monoclonal anti-PPARγ antibody (K8713, Perseus Proteomics) and analyzed by SDS-PAGE. Western blotting was carried out using a rabbit polyclonal anti-PPARγ antibody (H-100, Santa Cruz Biotechnology).

### Adenovirus construction and expression

Recombinant type 5 adenoviruses (Ad5) expressing GFP alone or with either WT or C114R mutant PPARγ1 were generated using the *AdEasy Vector System* (Quantum Biotechnologies, Montreal), amplified and purified as described (Gurnell et al., 2000). 6-well plates of Chub-S7 human preadipocyte cells were cultured and infected with 2x10^7^ pfu/well of recombinant virus 24 hr prior to differentiation in the presence of 100nM rosiglitazone as described (Darimont et al., 2003). Comparable infection efficiency was verified by fluorescence microscopy with subsequent qPCR analysis on days 0, 3, 5 and 7. Fully differentiated cells were fixed and stained with Oil Red-O as described (Adams et al., 1997).

## Figures and Tables

**Table 1:** Clinical, biochemical, and body composition details

Subject (gender)	S1 (F)	S2 (F)	S3 (F)	S4 (F)	S5 (F)
Mutation	C114R	C131Y	C162W	FS315X	R357X
Age (and at presentation, year)	41 (34)	42 (35)	31 (19)	13 (8)	35 (26)
BMI (kg/m^2^) (nonobese < 30)	30.0	24.2	30.5	25.9	29.3
BP (mmHg) (< 130/85)	155/95	220/120	150/100^∗^	125/65	125/80^∗^
T2DM/IGT (age at diagnosis, yr)	T2DM (41)	T2DM (42)	IGT (29)	T2DM (8)	T2DM (26)
PCOS	Y	Y	Y	N/A	Y
Hepatic steatosis	Y	Y	Y	Y	Y
TG (mmol/L) (<1.7)	8.9^∗^	4.5	5.0^∗^	8.3^∗^	34.8^∗^
HDL-C (mmol/L) (>1.29)	0.47^∗^	0.89	0.71^∗^	0.72^∗^	0.56^∗^
FI (pmol/L) (<60)	310	174	220^∗^	475^∗^	170^∗^
Predicted total body fat (%)	37.4	28.8	38.1	31.3	36.4
Measured total body fat (%)	26 ^−0.8^	23 ^−1.2^	nd	26 ^nd^	21 ^−1.1^
Measured lower limb fat (%)	20	17	nd	21	11
Measured truncal fat (%)	30	27	nd	31	28

BMI, body mass index; BP, blood pressure; T2DM, type 2 diabetes mellitus; IGT, impaired glucose tolerance; PCOS, polycystic ovarian syndrome; TG, triglycerides; HDL-C, high-density lipoprotein cholesterol; FI, fasting insulin; Predicted total body fat was calculated as follows (Black et al., 1983): males % fat = (1.281 × BMI) − 10.13: females % fat = (1.48 × BMI) − 7.00; measured total and depot-specific body fat were determined using dual-energy X-ray absorptiometry, with corresponding z scores for total body fat shown as superscript; Hepatic steatosis was diagnosed according to standard radiological criteria; F, female; healthy adult values where available are shown in parentheses; asterisk denotes patient studied on treatment; N/A, not applicable; nd, not determined.

## Supplemental data

Document S1. Supplemental experimental procedures and three figures
