# Supplementary material for: Non-DNA binding, dominant-negative, human PPARγ mutations cause lipodystrophic insulin resistance
Source: Cell Metab. 2006 Oct;4(4):303–11. doi: 10.1016/j.cmet.2006.09.003 (PMC1821092; doi:10.1016/j.cmet.2006.09.003)
Supplement: Document S1. Supplemental experimental procedures and three figures [file mmc1.pdf]

Cell Metabolism, Volume *4*

Supplemental data

Non-DNA binding, dominant-negative, human PPARγ mutations cause lipodystrophic insulin resistence

Maura Agostini, Erik Schoenmakers, Catherine Mitchell, Istvan Szatmari, David Savage, Aaron Smith, Odelia Rajanayagam, Robert Semple, Jian Luan, Louise Bath, Anthony Zalin, Mourad Labib, Sudhesh Kumar, Helen Simpson, Dirk Blom, David Marais, John Schwabe, Ines Barroso, Richard Trembath, Nicholas Wareham, Laszlo Nagy, Mark Gurnell, Stephen O'Rahilly, and Krishna Chatterjee

Supplemental experimental procedures

Case Histories of Subjects

Subject 1 (S1), presented at age 34yrs with oligomenorrhoea and subfertility, when dyslipidaemia was found. At age 41, partial lipodystrophy was noted; type 2 diabetes was diagnosed and diet-treated. She has developed severe three vessel coronary artery disease that was not alleviated by percutaneous revascularisation and is on triple antianginal therapy. She is heterozygous for a cysteine to arginine mutation at codon 114 (C114R) in PPARγ1 and her mother and sister are genetically unaffected with normal biochemistry; her father (genotype unknown) died age 60yrs from a myocardial infarction.

Subject 2 (S2), presented at age 35yrs with hypertension and syncopal episodes secondary to hyperinsulinaemia. Partial lipodystrophy and dyslipidaemia were noted and polycystic ovarian syndrome (PCOS) was diagnosed based on oligomenorrhoea and pelvic ultrasound appearances. At age 42, she developed type 2 diabetes which is diet controlled; although a non-smoker, she has developed single vessel coronary artery disease age 44yrs. She is heterozygous for a cysteine to tyrosine mutation at codon 131 (C131Y) of PPARγ1. A genetically affected younger sister is insulin resistant and

dyslipidaemic (fasting insulin [FI] 168 pmol/L; triglycerides [TG] 8.6mmol/L, high density lipoprotein cholesterol [HDL-C] 1.0mmol/L), whereas an unaffected older sister is biochemically normal (FI 79 pmol/L; TG 0.8mmol/L, HDL-C 1.4mmol/L). Her genetically affected father was a long-term smoker and deceased from lung carcinoma.

Subject 3 (S3), presented at age 19yrs with eruptive xanthomata secondary to severe hypertriglyceridaemia. Retrospectively, partial lipodystrophy was present since age 8, PCOS was diagnosed in her twenties and hypertension and acanthosis nigricans together with impaired glucose tolerance were noted age 29yrs. She is heterozygous for a cysteine to tryptophan mutation at codon 162 (C162W) in PPARγ1. Significant hypertriglyceridaemia (TG 26mmol/L) has been diagnosed since age 49yrs in her genetically affected mother together with hypertension, type 2 diabetes and ischaemic heart disease age 52yrs; her genetically affected grandfather has type 2 diabetes and ischaemic heart disease.

Subject 4 (S4), presented at age 8yrs with diabetes mellitus and partial lipodystrophy, acanthosis nigricans and severe hypertriglyceridaemia with eruptive xanthomata were noted; she is currently on metformin, pioglitazone and insulin therapy. She is heterozygous for a frameshift mutation predicting a premature stop mutation at codon 315 ([$A^{935}\Delta C$]fs.312[stop315]; FS315X) in PPARγ1. Her mother is known to have developed type 2 diabetes aged 16 and possible hypertension but is deceased and of unknown genotype. Her maternal grandfather is genetically unaffected and family members are untraceable.

Subject 5 (S5), presented at age 26yrs with gestational diabetes and hypertension and pregnancy was complicated by preeclampsia. Type 2 diabetes and hypertension persisted

post-partum and dyslipidaemia was noted subsequently leading to episodes of pancreatitis. Partial lipodystrophy and acanthosis nigricans were present. Treatment includes metformin, fenofibrate and insulin. She is heterozygous for a mutation changing arginine at codon 357 to a stop mutation (R357X). Her deceased mother, who was found to be genetically affected retrospectively, developed hypertension in her thirties, type 2 diabetes and dyslipidaemia (TG 4.9mmol/L, HDL-C 0.6mmol/L) in her forties and died suddenly from cardiovascular cause age 57.

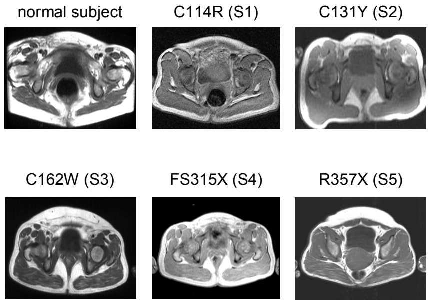

Figure S1. T1-weighted MRI scans at the level of the gluteal fat pad in PPARγ mutation carriers and a gender-matched healthy control subject. Note the striking diminution of the gluteal fat depot in all probands (S1-S5) with PPARγ mutations.

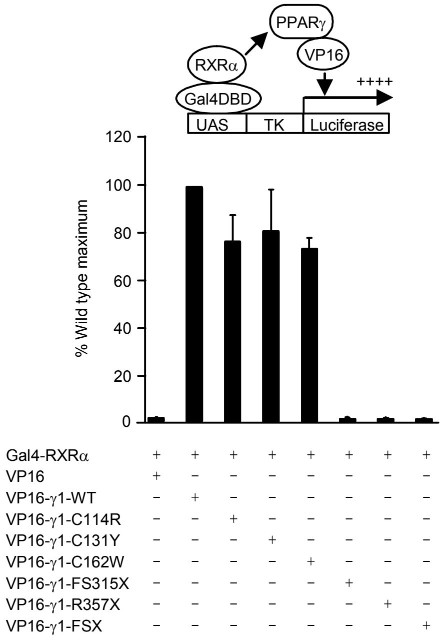

Figure S2. PPARγ mutants differ in their ability to interact with RXR. In a mammalian 2-hybrid assay, the DNA-binding domain mutants (C114R, C131Y and C162W) are recruited to RXR comparably to WT, whereas the FS315X, R357X and FSX truncation mutants, which lack an RXR interaction domain, exhibit negligible interaction. 293EBNA cells were transfected with 500ng of UASTKLUC reporter construct, 100ng of the internal control Bos-β-gal, 50ng of Gal4DBD-RXRα and 50ng of expression vector encoding either VP16 alone or VP16-full length WT or mutant PPARγ fusions. Results are expressed as a percentage of the WT maximum response and represent the mean ± s.e.m. of at least 3 independent experiments, each performed in triplicate.

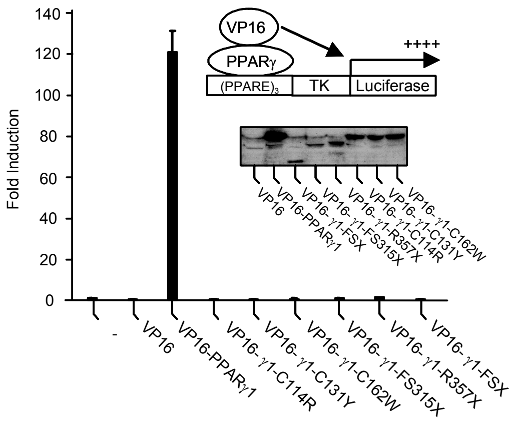

Figure S3. PPARγ mutants fail to bind to DNA. Chimaeric fusion proteins consisting of the VP16 activation domain linked to the N-terminus of full-length PPARγ1 (WT or mutant) were co-expressed in 293EBNA cells with a PPARE-containing reporter gene [(PPARE)$_3$TKLUC]. Interaction of WT VP16-PPARγ with (PPARE)$_3$TKLUC markedly increased transactivation. In contrast, levels of reporter gene activity in cells expressing mutant chimaeras were similar to mock-transfected cells, suggesting no significant promoter interaction. 96-well plates of 293EBNA cells were transfected with 9ng of (PPARE)$_3$TKLUC, 1.6ng of Bos-β-gal, and 1.6ng of the respective VP16-PPARγ1 chimaeras as shown. Inset, ^{35}S-labeled *in vitro* translated wild type and mutant VP16-PPARγ fusion proteins. Results are expressed as fold induction relative to cells transfected with VP16 alone and represent the mean ± s.e.m. of at least three independent experiments, each performed in triplicate.
